# Prognostic impact of Ikaros expression in lenalidomide-treated multiple myeloma

**DOI:** 10.18632/oncotarget.22572

**Published:** 2017-11-21

**Authors:** Jan Krönke, Stefan Knop, Christian Langer

**Affiliations:** Jan Krönke: Department of Internal Medicine III, Ulm University Hospital, Ulm, Germany

**Keywords:** lenalidomide, ikaros, gene expression, multiple myeloma, prognostic marker

Multiple Myeloma is a malignant plasma cell disorder characterized by the development of end organ damages resulting in hypercalcemia, renal insufficiency, anemia and osteolytic lesions. The therapeutic options for multiple myeloma have markedly increased over the last decade. In particular, the development of proteasome inhibitors like bortezomib and carfilzomib and the immunomodulatory agents (IMiDs) thalidomide, lenalidomide and pomalidomide have become the cornerstones of current treatment strategies.

In the past years, a distinct mode of action for lenalidomide and its analogs thalidomide and pomalidomide has been revealed. These compounds induce degradation of the transcription factors Ikaros (IKZF1) and Aiolos (IKZF3), that are essential for myeloma cell proliferation, via the E3 ubiquitin ligase complex CRL4 (CRBN) [1-3]. More recently, another CRBN-dependent mechanism that results in destabilization of Basigin (BSG) and monocarboxylate transporter 1 (MCT-1) has been described for the activity of lenalidomide in multiple myeloma [4].

In order to determine whether expression of genes involved in the mechanism of lenalidomide has an impact on outcome, we measured RNA expression levels of *CRBN, IKZF1, IKZF3* and *BSG* in pretreatment samples of patients treated with a lenalidomide-comprising regimen [5]. Patients up to the age of 65 years where treated on one of the up-front protocols of the German multiple myeloma study group (DSMM XII protocol) to receive 4 cycles of a combination of leanlidomide, doxorubicin and dexamethasone (RAD) followed by an intensive consolidation strategy including an autologous stem cell transplantation and in selected high risk cases an allogenic transplantation. All patients were scheduled to also receive a lenalidomide maintenance therapy.

We observed a trend for higher *IKZF1* expression levels in patients with International Staging System (ISS) stage III compared with ISS stage I and II. Patients who achieved a complete response or very good partial response at the time point of best response showed a trend towards lower pretreatment *IKZF1* expression levels compared with patients with a partial response or stable disease. Using mRNA expression levels as continuous variables in univariate Cox regression analysis, we found that high *IKZF1* levels were associated with an adverse PFS. Multivariable Cox regression analysis including genetic aberrations of known prognostic impact like t(4;14) and del(17p) revealed that pretreatment *IKZF1* expression levels are an independent marker for PFS. When we divided the groups by quartiles of *IKZF1* expression, patients within the lowest quartile had an excellent outcome with an estimated 3-year PFS of 86% and an estimated 3-year OS rate of 100% as compared to patients in the higher quartiles (3-year PFS 51%, OS 74%). In the subgroup of cytogenetically defined standard-risk patients *IKZF1, IKZF3,* and *BSG* expression levels had a significant impact on PFS.

**Figure 1:**
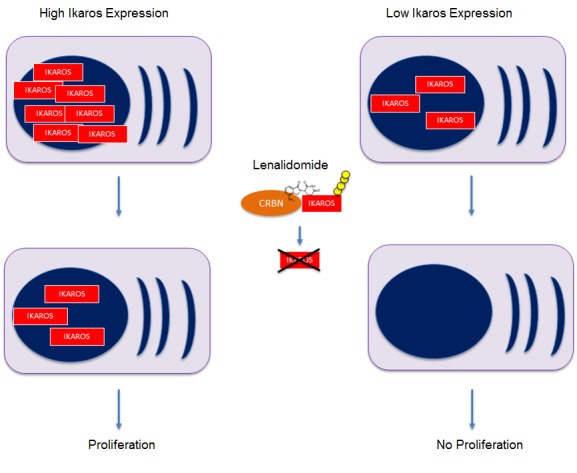
Potential model for reduced lenalidomide-sensitivity of Multiple Myeloma cells with high expression of CRBN target proteins like Ikaros, Aiolos and BSG High protein amounts prevent lenalidomide-induced degradation to critical low levels in the cells.

Our results suggest that multiple myeloma cells with low Ikaros expression are more sensitive to therapy. This may be counterintuitive on the first sight when considering a model where high expression of a gene indicates that it is relevant for the tumor cell and driving proliferation. However, almost all treatment-naïve multiple myeloma patients are primarily sensitive to lenalidomide and dexamethasone, suggesting that myeloma cells are in general Ikaros-dependent. Consistent with in vitro data in multiple myeloma cell lines, high expression of Ikaros, Aiolos and BSG may prevent lenalidomide-induced degradation of these proteins to critical low levels in those cells with high expression [1, 2, 4] (Figure 1). However, since the patients in our cohort received dexamethasone as well as doxorubicin and high-dose melphalan, we cannot rule out that high Ikaros expression is a general marker for therapy resistance to multiple treatments. Previous studies with pomalidomide in relapsed/refractory patients came to opposing results, showing that those patients with high Ikaros expression are more sensitive [3, 6]. This may be explained by 1) that multiple myeloma relapsed after or refractory to lenalidomide may not necessarily be Ikaros dependent anymore and high Ikaros expression identifies those cases that still depend on Ikaros; 2) pomalidomide is more potent than lenalidomide and may overcome relative resistance by high Ikaros expression. Other studies did not see an impact of Ikaros expression on outcome [7, 8]. While *CRBN* expression levels were associated with outcome in other studies, there was no impact on PFS or OS in our cohort. The major differences between all studies that may explain for these different observations are the patient population (newly diagnosed vs. relapsed/refractory), treatment (pomalidomide or lenalidomide in combination with other drugs), and assays applied (RNA analysis by RQ-PCR or arrays, protein analysis by immunohistochemistry or FACS/immunofluorescence).

In order to determine the predictive and prognostic value of Ikaros and other cooperating members of the CRBN complex, future studies need to analyze gene and protein expression levels by standardized assays in larger patient cohorts treated with different protocols in controlled clinical trials. The identification of predictive markers for specific drugs like lenalidomide has the potential to further personalize treatment in multiple myeloma.
